# Alterations of the Brain Microstructure and Corresponding Functional Connectivity in Early-Blind Adolescents

**DOI:** 10.1155/2019/2747460

**Published:** 2019-02-24

**Authors:** Zhifeng Zhou, Jinping Xu, Leilei Shi, Xia Liu, Fen Hou, Jingyi Zhou, Jinpei Luo, Qingmao Hu, Hengguo Li

**Affiliations:** ^1^Department of Radiology, Shenzhen Mental Health Center, Shenzhen Kangning Hospital, Shenzhen 518003, China; ^2^Institute of Biomedical and Health Engineering, Shenzhen Institutes of Advanced Technology, Chinese Academy of Sciences, Shenzhen 518055, China; ^3^Medical Imaging Center, The First Affiliated Hospital of Jinan University, Guangzhou 510630, China; ^4^Department of Mechanical Engineering, Sungkyunkwan University, Suwon 16419, Republic of Korea

## Abstract

Although evidence from studies on blind adults indicates that visual deprivation early in life leads to structural and functional disruption and reorganization of the brain, whether young blind people show similar patterns remains unknown. Therefore, this study is aimed at exploring the structural and functional alterations of the brain of early-blind adolescents (EBAs) compared to normal-sighted controls (NSCs) and investigating the effects of residual light perception on brain microstructure and function in EBAs. We obtained magnetic resonance imaging (MRI) data from 23 EBAs (8 with residual light perception (LPs), 15 without light perception (NLPs)) and 21 NSCs (age range 11-19 years old). Whole-brain voxel-based analyses of diffusion tensor imaging metrics and region-of-interest analyses of resting-state functional connectivity (RSFC) were performed to compare patterns of brain microstructure and the corresponding RSFC between the groups. The results showed that structural disruptions of LPs and NLPs were mainly located in the occipital visual pathway. Compared with NLPs, LPs showed increased fractional anisotropy (FA) in the superior frontal gyrus and reduced diffusivity in the caudate nucleus. Moreover, the correlations between FA of the occipital cortices or mean diffusivity of the lingual gyrus and age were consistent with the development trajectory of the brain in NSCs, but inconsistent or even opposite in EBAs. Additionally, we found functional, but not structural, reorganization in NLPs compared with NSCs, suggesting that functional neuroplasticity occurs earlier than structural neuroplasticity in EBAs. Altogether, these findings provided new insights into the mechanisms underlying the neural reorganization of the brain in adolescents with early visual deprivation.

## 1. Introduction

It is well established that early-blind adults, i.e., those individuals who lose sight at birth or within a short period after birth, show alterations in the neural structure and function due to the absence of visual inputs. Structurally, the morphological changes of the grey matter (GM) in early-blind adults have been studied extensively and include decreased GM volume in the primary visual area [1–5]; increased GM volume in the occipital, frontal, and entorhinal cortices [4] and the globus pallidus [6]; and increased cortical thickness of the early visual areas [2, 7, 8]. In addition to GM alterations, white matter (WM) alterations were also identified based on the diffusion tensor imaging (DTI) datasets of early-blind adults. In detail, microstructural alterations were mainly found in the optic radiation and the lateral geniculate nucleus with decreased WM volume [2, 9, 10] or density [9, 11]. Other microstructural disruptions due to early visual deprivation were also found in the inferior longitudinal fasciculus [12] and the occipital/temporal/parietal thalamocortical projections [4, 13]. In addition, Leporé et al. [3], using tensor-based morphometry, reported interesting hypertrophy in the prefrontal and parietal WM and in a section of the splenium of the corpus callosum in early-blind adults. Interestingly, some studies [11, 14] also identified increased fractional anisotropy in the corticospinal tract in early-blind adults by tract-based quantitative analysis, suggesting structural neuroplasticity. To some extent, such morphological changes reflect deprivation-induced processes, both neurodegenerative and neurodevelopmental, following the loss of visual input and/or altered visual experience [3, 5].

In addition to structural alterations, functional changes were also identified in early-blind adults in many studies. Task-based research using functional magnetic resonance imaging (fMRI) demonstrated that the occipital cortex could process nonvisual information, such as tactile [15, 16] and auditory input [16–20], and some complex cognitive- and verbal-related input [17, 21–23]. These results collectively suggested that the visual cortex of blind individuals exhibits cross-modal reorganization properties. Moreover, activity in the primary, secondary, and tertiary visual cortices of blind adults was increased, spatially and in amplitude, after sensory substitution training, even in short-term (10 min) [24]. Using resting-state fMRI (rs-fMRI), both decreased functional connectivity [7, 22, 25–28] and increased functional connectivity of the occipital cortex [25, 29, 30] were identified in early-blind adults compared with sighted controls, providing extra evidence of functional neuroplasticity in early-blind adults.

However, these results about structural and functional disruptions and neuroplasticity were derived from early-blind adults. Recently, we investigated resting-state interhemispheric functional connectivity in early-blind adolescents (EBAs) and found significantly lower voxel-mirrored homotopic connectivity (VMHC) in the primary visual cortex, visual association cortex, and somatosensory association cortex [31]. Although the study focused on EBAs, it is still unclear if this group would show structural and functional alteration patterns similar to those of early-blind adults. Moreover, most of the previous studies on early blindness included subjects with residual light perception, thus failing to exclude possible effects of residual light perception on the structure and function of the blind brain. Therefore, we aimed to (1) investigate whether and how the brain's structure and function are altered in EBAs and (2) investigate the effects of residual light perception on brain structure and function. Given the evidence just discussed, we hypothesized that structural and functional alterations in EBAs would mostly be identified in the brain regions associated with vision. To verify this hypothesis, we recruited a cohort of 23 EBAs (8 with residual light perception (LPs), 15 without light perception (NLPs)) and 21 NSCs (age range: 11–19 years old) to explore the structural and functional brain changes in EBAs using the whole-brain voxel-based analysis (VBA) and the resting-state functional connectivity (RSFC).

## 2. Materials and Methods

### 2.1. Subjects

Twenty-three EBAs with onset age < 1 year were enrolled from the Guangzhou City Blind School. Among them, 8 EBAs with residual light perception were included in the LP group (6 males and 2 females) and 15 EBAs without light perception were included in the NLP group (8 males and 7 females). Twenty-one age- and sex-matched normal-sighted volunteers were recruited as the NSC group (10 males and 11 females). The demographics of the three groups are summarized in Table 1. All subjects met the following inclusion criteria: (1) right-handed and (2) age ranging from 11 to 19 years. The exclusion criteria were (1) any history of psychiatric or neurologic diseases, (2) symptomatic or atypical neuralgia, and (3) identifiable MRI abnormalities, such as demyelination, vascular malformations, or tumors. All individuals and their guardians signed a written informed consent form prior to the MRI examinations. All research procedures were approved by the Ethics Committee of the First Affiliated Hospital of Jinan University.

### 2.2. Data Acquisition

A 3.0T MRI scanner (Discovery MR750 System; General Electric, Milwaukee, WI, USA) with an 8-channel head coil was used. The subjects were scanned in the supine position and were placed head first into the scanner. The position of the head was fixed using several foam cushions. For each subject, the DTI and rs-fMRI data were acquired. For DTI, we acquired 75 diffusion-weighted images (*b* = 1000 s/mm^2^) and 5 nondiffusion-weighted images (*b* = 0 s/mm^2^) using a spin-echo echo-planar imaging sequence with the following parameters: number of excitations = 1, repetition time (TR) = 6000 ms, echo time (TE) = 68 ms, acquisition matrix = 128 × 128, and voxel size = 2 mm × 2 mm × 3 mm. The array spatial sensitivity encoding technique was used with an acceleration factor of 2 to reduce acquisition time and anamorphosis. The rs-fMRI data were acquired using an echo-planar imaging pulse sequence, sensitive to blood-oxygen-level-dependent contrast (TR = 2000 ms, TE = 35 ms, flip angle = 90°, matrix size = 64 × 64, field of view = 256 × 256 mm^2^, slice thickness = 3 mm, gap = 0.6 mm, 240 time points, sequence length = 8 min, and 41 slices in the axial plane). During rs-fMRI, subjects were instructed to stay awake, not to move, to think of nothing in particular, to relax, and to keep their eyes closed.

### 2.3. VBA and Statistics Analysis

We performed DTI preprocessing using the FMRIB Software Library (FSL5.0; http://www.fmrib.ox.ac.uk/fsl), including eddy-current and motion correction, brain mask creation for the DTI data, exclusion of voxels outside the braincase, and diffusion tensor reconstruction with the “DTIFIT” toolbox [10]. All diffusion-related parameters including fractional anisotropy (FA), mean diffusivity (MD), radial diffusivity (RD), and axial diffusivity (AD) were calculated. Following this, individual FA maps were analyzed using the statistical parametric mapping software (SPM8; Wellcome Department of Cognitive Neurology, London, UK; http://www.fil.ion.ucl.ac.uk/spm). The FA maps were normalized to the standard Montreal Neurological Institute (MNI) template and smoothed with a 6 mm full-width at half-maximum (FWHM) Gaussian kernel to reduce the effects of misregistration in spatial normalization [32]. Two-sample *t*-tests were conducted to assess intergroup differences in FA between the groups with age and sex as covariates. In order to account for multiple comparisons, the AlphaSim method was used with a voxel-level threshold of *p* < 0.001 and a cluster-level threshold of *p* < 0.05. VBA of the MD, RD, and AD maps was performed similarly.

The clusters showing significant differences in the DTI parameters between the groups were identified according to the standard brain atlas, and the DTI values from these significant clusters of the corresponding tensor parameter maps (FA, MD, RD, and AD) in the subjects were extracted. Subsequently, correlation analysis followed by an independent two-sample *t*-test was performed using the Statistical Package for the Social Sciences (SPSS) software (version 23.0; IBM Corporation, NY, USA). For correlations between DTI metric values and age, the Pearson correlation coefficient was computed separately for each group. Two-sample *t*-tests were used to detect differences in the DTI values between the groups.

### 2.4. Resting-State fMRI Data Preprocessing

The rs-fMRI data were preprocessed using the toolbox for Data Processing & Analysis for Brain Imaging (DPABI, http://rfmri.org/dpabi) [33]. The preprocessing steps were (1) discarding the first 10 time points to avoid transient signal changes that occurred before magnetization reached the steady state and subjects' adaptation to the scanning noise; (2) slice timing correction; (3) head motion realignment; (4) normalization to the standard MNI template in the DPABI package; (5) smoothing with a 6 mm Gaussian kernel; (6) linear regression to remove confounding factors, including six head motion parameters, as well as the ventricle, WM, and global signals; (7) scrubbing with interpolation to remove volumes with head motion spatial shift larger than 1.5 mm and rotation greater than 1.5° in any direction; and (8) band-pass filtering from 0.01 to 0.1 Hz. The resulting images were manually inspected by two experienced researchers for image quality.

### 2.5. RSFC and Statistics Analysis

To investigate the corresponding functional alterations, the clusters showing significant group difference in VBA were selected as the seed ROIs for RSFC analysis. Firstly, the peak coordinates of each significant cluster obtained from VBA were determined and used to create a ROI with a radius of 6 mm. Secondly, the RSFC was examined with a seed-voxel correlation approach, in which the correlations of the mean time-course signal in a seed region with all other voxels in the whole brain were calculated. Before statistical analysis, the correlation coefficients were transformed into *z* values using the Fisher *r*-to-*z* transformation to improve the normality. Two-sample *t*-tests between the groups were then performed using the DPABI toolbox, with age, sex, and head motion parameters as covariates. The statistical results were corrected for multiple comparisons using the AlphaSim method, with a voxel-level threshold of *p* < 0.001 and a cluster-level threshold of *p* < 0.05. Finally, the results were projected onto a smoothed International Consortium for Brain Mapping (ICBM152) surface template using the BrainNet Viewer (http://www.nitrc.org/projects/bnv/) [34].

## 3. Results

### 3.1. Demographic Characteristics

Six male and two female subjects were included in the LP group and eight male and seven female subjects in the NLP group; we also recruited 21 age- and sex-matched NSCs. Analysis of variance (ANOVA) followed by two-sample *t*-tests and chi-square tests were performed to detect age and sex differences, respectively, between the various groups (Table 2). No significant differences in the demographic characteristics were identified between the three groups (age: *t* = −1.657, *p* = 0.112 comparing the LP and NLP groups; *t* = −1.883, *p* = 0.071, LP vs. NSC; *t* = 0.271, *p* = 0.788, NLP vs. NSC; sex: *p* = 0.400, LP vs. NLP; *p* = 0.238, LP vs. NSC; and *p* = 1.000, NLP vs. NSC).

### 3.2. VBA Results

**Figure 1:**
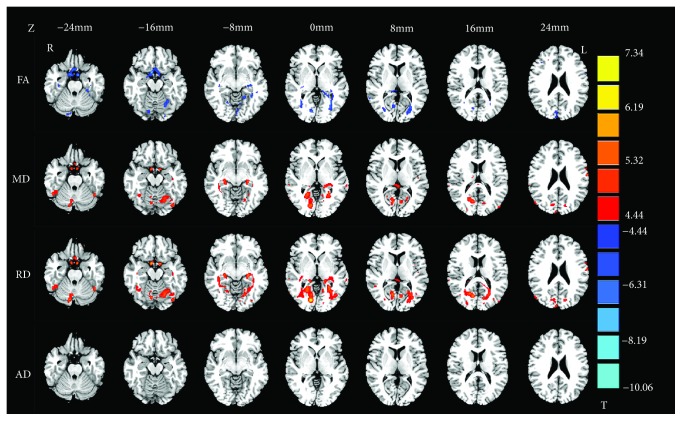
Group differences of fractional anisotropy (FA), mean diffusivity (MD), radial diffusivity (RD), and axial diffusivity (AD) in NLPs compared to NSCs (*p* < 0.0001, AlphaSim corrected). Blue regions denote lower FA values and red regions denote higher diffusivity values. The numbers at the top indicate the *z* value of MNI coordinates.

**Figure 2:**
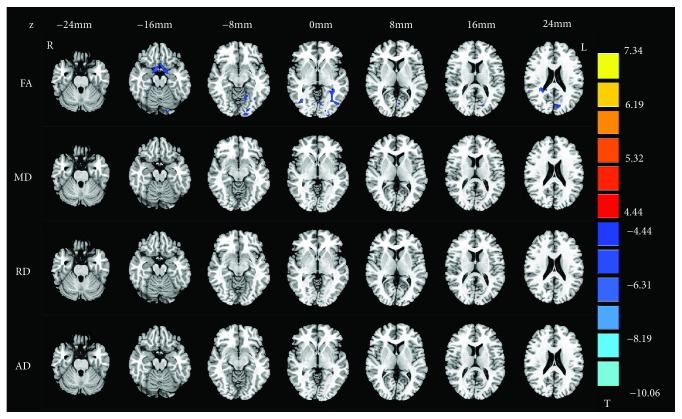
Group differences of fractional anisotropy (FA), mean diffusivity (MD), radial diffusivity (RD), and axial diffusivity (AD) in LPs compared to NSCs (*p* < 0.001, AlphaSim corrected). Blue and red regions denote lower and higher DTI parameters, respectively. The numbers at the top indicate the *z* value of MNI coordinates.

The two-sample *t*-test revealed a reduced FA in the left occipital lobe/subgyral, right parahippocampal gyrus, and right superior occipital gyrus in NLPs compared to NSCs. We also found higher MD in the bilateral lingual gyrus, as well as higher RD in the right lingual gyrus and right parahippocampal gyrus in NLPs, compared with NSCs (*p* < 0.001, AlphaSim corrected; Table 3 and Figure 1). No significant AD alterations were observed between these two groups. Compared with NSCs, LPs showed significant FA reduction in the bilateral middle occipital gyrus, left lingual gyrus, left cuneus, left parahippocampal gyrus, and right parietal lobe/subgyral (*p* < 0.001, AlphaSim corrected; Table 3 and Figure 2). No significant differences in MD, RD, and AD were found when comparing the LP and NSC groups. No elevated FA or reduced diffusivity parameters were found for any of the clusters in NLPs or LPs compared with NSCs, even with a loose threshold (*p* < 0.01, AlphaSim corrected). Unfortunately, the comparison of all diffusion tensor maps between the LP and NLP groups showed no results at the voxel level, at AlphaSim-corrected *p* < 0.001. However, some voxels in the brain of the LP group exhibited trends (at a voxel-level AlphaSim-corrected *p* < 0.01) towards increased/decreased DTI parameters compared with NLPs, including reduced FA in the right fusiform gyrus, right middle temporal gyrus, and superior occipital gyrus; increased FA in the right superior frontal gyrus; increased MD, RD, and AD in the right fusiform gyrus and middle temporal gyrus; and reduced MD, RD, and AD in the bilateral caudate (Table SI and Figure SI in the Supplementary Materials).

### 3.3. Correlation Analysis Results

**Figure 3:**
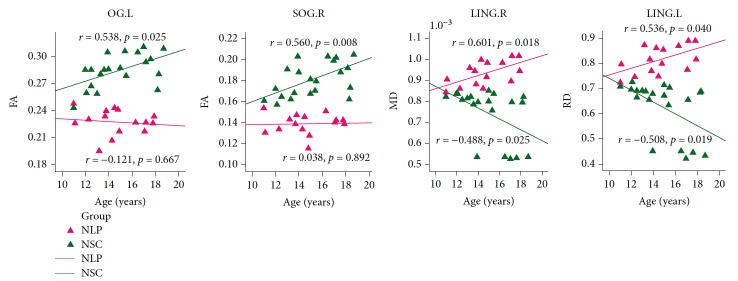
Pearson's correlation analyses between age and DTI metrics of OG.L, SOG.R, LING.R, and LING.L, respectively, in the NLP and NSC groups. In the scatter plots, the DTI metrics of each significant region are displayed for each subject in the specific groups, with dots for NLPs shown in violet and NSCs in green. The correlation coefficients and *p* values are next to each scatter plot. All abbreviations of brain regions are shown in Table 3.

Correlation analysis showed positive and significant correlations between the MD and RD values of the right lingual gyrus and age in NLPs (MD: *r* = 0.601, *p* = 0.018; RD: *r* = 0.536, *p* = 0.040), but negative and significant correlations in NSCs (MD: *r* = −0.488, *p* = 0.025; RD: *r* = −0.508, *p* = 0.019). In NSCs, the FA values of the left occipital lobe/subgyral (*r* = 0.538, *p* = 0.012) and the right superior occipital gyrus (*r* = 0.560, *p* = 0.008) were positively correlated with age, while the MD values of the left lingual gyrus were negatively correlated with age (*r* = −0.508, *p* = 0.019) (Figure 3).

### 3.4. Head Motion Effects

Head motion time courses were computed by estimating the shift in each direction and the rotations about each axis for each of the 230 consecutive volumes. To assess the head motion confounders, we calculated the mean framewise displacement among the three groups (LP: 0.35 ± 0.23 mm; NLP: 0.22 ± 0.17 mm; and NSC: 0.26 ± 0.27 mm). Moreover, ANOVA was used to identify potential differences in head motion between the 3 groups, but none was found. (ANOVA: *F* = 0.845, *p* = 0.437).

### 3.5. RSFC Results

**Figure 4:**
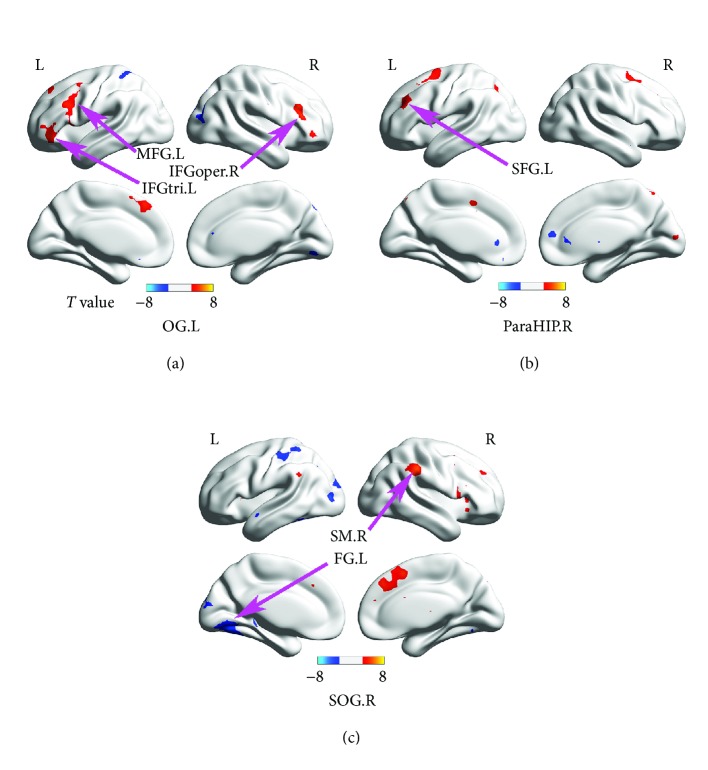
Brain regions that showed altered functional connectivity with OG.L (a), ParaHIP.R (b), and SOG.R (c), respectively, in NLPs compared to NSCs. Two-sample *t*-tests were performed to explore the between-group differences. The results were corrected by the AlphaSim method at a voxel level of *p* < 0.001. All abbreviations of the brain regions are shown in Tables 3 and 4.

The whole-brain RSFC of the brain regions with significant microstructural alterations was analyzed and compared between the NLP and NSC groups. The left occipital lobe/subgyral of NLPs showed enhanced RSFC with the left inferior frontal triangular areas, left middle frontal gyrus, and right inferior frontal opercular areas; the right parahippocampal gyrus of NLPs showed enhanced RSFC with the left superior frontal gyrus; the right superior occipital gyrus of NLPs showed enhanced RSFC with the right supramarginal gyrus/left superior frontal gyrus and decreased RSFC with the left fusiform gyrus (*p* < 0.001, AlphaSim corrected; Table 4 and Figure 4). When comparing LPs with NSCs, only the left middle occipital gyrus showed a significantly decreased RSFC in LPs with the right middle occipital gyrus, when setting the bilateral middle occipital gyrus, left parahippocampal gyrus, left lingual gyrus, left cuneus, and right parietal lobe/subgyral as ROI seeds (*p* < 0.001, AlphaSim corrected; Table 4).

To investigate the effects of residual light perception on the brain function in blind brains, the differences in the whole-brain RSFC of the brain regions showing microstructural alterations between the LP and NLP groups were analyzed and compared. A trend of RSFC change in LPs relative to NLPs could also be observed and specifically enhanced RSFC between the right fusiform gyrus and the right superior frontal orbital areas/right middle temporal gyrus and between the right superior frontal gyrus and the left inferior temporal gyrus. On the contrary, the RSFC between the right superior frontal gyrus and the right postcentral gyrus/superior frontal gyrus was decreased in the LP compared to the NLP group (*p* < 0.01, AlphaSim-corrected; Table SII and Figure SII in the Supplementary Materials).

## 4. Discussion

We investigated the alterations of brain microstructure and their corresponding functional connectivity in EBAs with and without light perception using the DTI and rs-fMRI datasets. Compared to NSCs, both EBA groups showed significant brain microstructural disruptions mainly in the posterior visual pathway of the occipital lobe and the extrastriate visual cortex. Moreover, the correlations between bilateral occipital cortex FA or bilateral lingual gyrus MD and age were consistent with the developmental trajectory of the brain in NSCs, but inconsistent or even opposite in EBAs. Additionally, using brain regions with significant DTI parameter changes in our study as the ROIs, we identified enhanced RSFC in the occipital cortex with the bilateral frontal cortex and the homolateral parietal cortex and reduced intrahemispheric RSFC in the right visual cortex in NLPs, as well as reduced interhemispheric RSFC within the middle occipital cortex in LPs compared with NSCs. Our findings provide additional evidence that early visual deprivation may lead to functional neuroplasticity earlier than structural neuroplasticity in EBAs.

### 4.1. Disruptions in the Brain Microstructure of EBAs

The FA reduction in the bilateral visual pathway of the occipital cortex in both EBA groups, elevated MD of the bilateral lingual gyrus, and elevated RD of the right lingual gyrus in NLPs vs. NSCs were consistent with the findings of previous research on young or middle-aged blind adults [2, 9, 12, 35, 36]. These structural disruptions were also supported by other evidence of reduced anatomical connectivity associated with the visual cortex [12, 37] and decreased GM and WM volume in the occipital cortex of blind individuals [6, 38]. Our study also validated the GM and WM changes in the visual cortex of EBAs at the microstructural level, which can be explained by transneuronal degeneration and/or immaturity due to early visual deprivation [9, 39]. Moreover, we found diminished FA in the left parahippocampal gyrus of LPs and the right parahippocampal gyrus of NLPs compared to NSCs. The right parahippocampal gyrus is important for learning and visuospatial configuration of objects [40], while the left parahippocampal gyrus is involved in cumulative verbal memory [41]. Considering the asymmetry function of the bilateral parahippocampal gyri during learning, their inconsistent alteration in the two groups may provide evidence of different learning patterns between the LP and NLP groups.

Furthermore, to investigate the effects of residual light perception on brain microstructure, we compared the DTI parameters between LPs and NLPs. The results showed increased FA in the right superior frontal gyrus and reduced diffusivity parameters in the bilateral caudate nucleus in LPs compared with NLPs. Since the superior frontal gyrus is anatomically connected with the cognitive control network and functionally involved in complex cognitive processing [42, 43], the increased FA of the right superior frontal gyrus in LPs vs. NLPs may suggest that residual light perception could play a role in complex cognitive function or reduce the damage to cognitive function induced in EBAs by the lack of visual information. As the caudate nucleus is known to integrate spatial information with motor behavior and is associated with some learning processing [44–46], its decreased diffusivity parameters in LPs vs. NLPs demonstrate that the function of guiding motor performance and learning in the caudate nucleus may be partly mediated by light perception.

We also calculated the correlations between age and DTI metrics of the altered brain regions in the three groups. The FA of the bilateral occipital cortex was positively correlated with age in NSCs but not in NLPs, and the MD and RD of the right lingual gyrus were negatively correlated with age in NSCs, but positively in NLPs. The trends of DTI parameters alteration with age in NSCs were consistent with the developmental trajectory of WM in the young [47–49], while these trends were not observed or were reversed in NLPs. This inconsistency suggests that microstructural disruptions caused by early visual deprivation in the corresponding brain regions may progressively accumulate in adolescents. Given the continuous development of WM in adolescence, a probably more reasonable explanation for the trend shown by the NLPs is the coexistence of blindness-induced damage and normal cerebral development.

### 4.2. Alterations in Functional Connectivity

Changes in brain microstructure preferentially point to abnormalities in brain functional connectivity [50, 51]. Resting-state fMRI can reflect the integrated features of intrinsic functional connectivity in the brains of visually impaired persons [27]. Many previous studies reported changes in RSFC between the occipital cortex and other sensory [7, 25–27, 52], somatosensory [25, 26, 28, 52, 53], and motor cortices [25, 26, 28] in early-blind adults. In the current study, the occipital gyrus in NLPs showed enhanced RSFC with some frontal areas, including the left inferior frontal triangle, left middle frontal gyrus, left superior frontal gyrus, and right inferior frontal opercular areas. Such findings are consistent with those of previous studies [54, 55]. In healthy humans, these frontal regions are classically considered as high-level areas related to language and cognition and involved in verbal fluency [56, 57], single word and motor speech production [58, 59], and error detection and imitation [60, 61]. Beyond these classical functions, they also participate in verbal working memory [62, 63]. In blind individuals, the occipital visual cortex and the frontal language and cognitive areas were activated when performing Braille reading tasks [64, 65]. These findings indicated that these frontal areas might play an important role, related to the language network, in the blind brain. Additionally, compared with sighted controls, early-blind participants had more extensive activity in the occipital visual areas and frontal language areas when performing a vibrotactile memory task [64, 65]. All evidence may explain why the RSFC between the occipital areas and frontal areas was increased in the NLPs. From another perspective, the elevated RSFC between the occipital visual areas and the frontal language regions can provide compelling evidence for the existence of functional neuroplasticity in NLPs.

We also observed reduced interhemispheric RSFC between the bilateral middle occipital gyrus in LPs and between the right superior occipital gyrus and the left fusiform gyrus in NLPs compared with NSCs, extending the findings of many previous studies [22, 25, 31, 66]. According to some published reports, such reduced interhemispheric communication may be interpreted as extensive abnormalities in WM integrity, particularly in the corpus callosum [67, 68]. Many existing studies found WM abnormalities in the splenium of the corpus callosum, thought to play a crucial role in the communication between the two cerebral hemispheres, in early-blind young, middle-aged, or elderly adults [3, 10, 35, 69, 70]. However, no WM structural abnormalities were found in the splenium of the corpus callosum in the present study. One possible reason may be that the posterior part of the corpus callosum continues to develop during adolescence and increases in density [47, 48, 71, 72], likely counteracting the neurodegenerative effects of visual deprivation [73]. Based on prior and present findings, we speculated that the RSFC changes in EBAs might result not only from a combination mechanism of general loss and complementary plasticity [25] but also from an antagonistic mechanism of neural development. Thus, our findings provide new information regarding the functional profiles that emerge in the early life of blind adolescents.

Furthermore, we evaluated the RSFC differences between the LP and NLP groups to investigate the differences in functional connectivity patterns depending on residual light perception. Compared with NLPs, LPs showed enhanced RSFC in the right fusiform with the homolateral middle temporal gyrus and reduced RSFC in the right superior frontal gyrus with the right postcentral gyrus. In sighted subjects, the fusiform and middle temporal gyri have been reported to be involved in the ventral and dorsal visual streams, respectively, and to be robustly activated during object shape/location detection and visual-motor tasks [74, 75]. There is no doubt that light perception is crucial for such processing. Therefore, the residual perception of light in LPs may account for the enhanced RSFC between these regions. Given that the postcentral gyrus is well-known as the primary somatosensory area and is associated with fine touch sense, the decreased RSFC of this area in LPs may suggest that the touch sense of early-blind individuals with residual light perception is not as strong as that of those without light perception.

### 4.3. Limitation

Our findings of the effects of residual light perception on brain microstructure and function in EBAs were derived at a loose level of statistical significance, presumably due to the small sample size. Therefore, these results should be interpreted with caution, and further studies with larger sample size will be needed to provide solid evidence of these phenomena.

## 5. Conclusion

The present study demonstrated significant microstructural and functional alterations in EBAs with and without residual light perception when compared to NSCs. Our findings provide additional evidence that early visual deprivation may lead to functional neuroplasticity earlier than structural neuroplasticity in EBAs. The structural alterations in EBAs contain complex mechanisms including general loss, complementary plasticity, and neural development. Collectively, these results provided new insights into the mechanisms underlying the reorganization of the brain in adolescents with early visual deprivation.

## Figures and Tables

**Table 1:** Clinical characteristics of early-blind adolescents and normal-sighted controls.

No.	Age (years)	Gender	Age of onset (months)	Causes of blindness
LP01	11.8	M	2	ROP
LP02	14.7	M	2	Cataract
LP03	14.4	M	0	CG; EA
LP04	11.0	M	0	CRL
LP05	12.3	M	3	ROP
LP06	12.5	F	8	ROP
LP07	13.8	M	0	CRL
LP08	15.0	F	0	CRL
NLP01	14.3	M	3	ROP
NLP02	16.3	M	2	ROP
NLP03	17.2	M	8	OT
NLP04	11.0	M	0	ROP
NLP05	13.8	F	1	ROP; EA
NLP06	17.9	F	5	Cataract; EA
NLP07	12.3	F	3	ROP
NLP08	17.1	F	0	ROP; OA
NLP09	17.8	M	0	ROP
NLP10	14.5	F	1	ROP
NLP11	14.8	M	4	ROP
NLP12	14.9	M	0	CRL
NLP13	11.1	F	3	ET; EE
NLP14	13.2	M	0	CRL
NLP15	13.7	F	0	ROP
NSC01	12.5	F	—	—
NSC02	12.0	M	—	—
NSC03	14.0	M	—	—
NSC04	17.2	M	—	—
NSC05	13.6	M	—	—
NSC06	12.5	M	—	—
NSC07	13.3	M	—	—
NSC08	18.3	F	—	—
NSC09	12.1	M	—	—
NSC10	18.2	F	—	—
NSC11	15.5	M	—	—
NSC12	16.5	M	—	—
NSC13	13.9	M	—	—
NSC14	18.7	F	—	—
NSC15	17.6	F	—	—
NSC16	17.0	F	—	—
NSC17	15.4	F	—	—
NSC18	15.0	F	—	—
NSC19	11.0	F	—	—
NSC20	15.0	F	—	—
NSC21	13.0	F	—	—

Abbreviations: LP: early-blind adolescents with light perception; NLP: early-blind adolescents without light perception; NSC: normal-sighted controls; M: male; F: female; ROP: retinopathy of prematurity; OT: oxygen toxicity; EA: eyeball atrophy; CRL: congenital retinal lesions: CG, congenital glaucoma; OA: optic atrophy; ET: eyeball tumor; and EE: eyeball extraction.

**Table 2:** Demographic characteristics of the recruited subjects.

	LP	NLP	NSC	ANOVA statistics	*p* value
Gender (M/F)	6/2	8/7	10/11	*F* = 0.857	0.432
Age (years)	13.2 ± 1.5	14.7 ± 2.3	14.9 ± 2.3	*F* = 1.782	0.181

Abbreviations: LP: early-blind adolescents with light perception; NLP: early-blind adolescents without light perception; NSC: normal-sighted controls; ANOVA: analysis of variance; M: male; F: female.

**Table 3:** Brain regions with significant changes in diffusion tensor imaging metrics.

Between-group comparison	DTI parameter changes	Localizations of peak voxels	Abbreviations	Cluster size (voxels)	*T* score	Peak MNI		
						*x*	*y*	*z*
NLP vs. NSC (*p* < 0.001, AlphaSim corrected)	FA reduction	Left occipital lobe/subgyral	OG.L	5196	-7.73	-32	-62	-2
		Right parahippocampal	ParaHIP.R	978	-10.06	12	2	-22
		Right superior occipital gyrus	SOG.R	255	-8.34	24	-92	32
	MD elevation	Left lingual	LING.L	3698	5.85	-28	-54	-4
		Right lingual	LING.R	3596	5.84	20	-54	2
	RD elevation	Right lingual	LING.R	4030	6.74	14	-80	0
		Right parahippocampal	ParaHIP.R	7113	7.07	10	4	-18
LP vs. NSC (*p* < 0.001, AlphaSim corrected)	FA reduction	Left parahippocampal	ParaHIP.L	319	-6.58	-12	2	-20
		Left middle occipital gyrus	MOG.L	198	-5.98	-26	-100	2
		Left occipital lobe/subgyral	OG.L	224	-5.71	-32	-68	-2
		Left lingual	LING.L	142	-5.11	-4	-74	-4
		Right middle occipital gyrus	MOG.R	62	-5.99	38	-70	0
		Left cuneus	CUN.L	154	-5.98	-8	-84	30
		Right parietal lobe/subgyral	PG.R	120	-6.65	32	-40	24

Abbreviations: LP: early-blind adolescents with residual light perception; NLP: early-blind adolescents without light perception; NSC: normal-sighted controls; FA: fractional anisotropy; MD: mean diffusivity; RD: radial diffusivity; and MNI: Montreal Neurological Institute.

**Table 4:** Brain regions with significant changes of RSFC between groups.

Between-group comparison	ROI seeds	Brain regions	Abbreviations	Cluster size (voxels)	*T* score	Peak MNI		
						*x*	*y*	*z*
NLPs vs. NSC	OG.L	Left inferior frontal gyrus, triangular part	IFGtri.L	77	5.12	-39	33	6
		Right inferior frontal gyrus, opercular part	IFGoper.R	115	6.77	57	21	33
		Left middle frontal gyrus	MFG.L	97	5.68	-45	18	18
	ParaHIP.R	Left superior frontal gyrus	SFG.L	67	5.96	67	-24	30
	SOG.R	Left fusiform gyrus	FG.L	188	-5.93	-24	-66	-15
		Right supramarginal gyrus	SMG.R	62	5.16	60	-33	39
		Left superior frontal gyrus	SFG.L	48	4.63	24	-93	6
LPs vs. NSC	MOG.L	Right middle occipital gyrus	MOG.R	47	-5.15	24	-93	6

The results were corrected by the AlphaSim method at a voxel level of *p* < 0.001. Abbreviations: RSFC: resting-state functional connectivity; ROI: region of interest; OG.L: left occipital gyrus; ParaHIP.R: right parahippocampal gyrus; SOG.R: right superior occipital gyrus; MOG.L; left middle occipital gyrus; LP: early-blind adolescents with residual light perception; NLP; early-blind adolescents without light perception; NSC: normal-sighted controls; MNI: Montreal Neurological Institute.

## Data Availability

The datasets generated and/or analyzed during the current study are available from the corresponding author Dr. Hengguo Li (lhgjnu@263.net) on reasonable request.

## Supplementary Materials

Supplementary Materials Table SI: brain regions with significant changes in diffusion tensor imaging metrics between LPs and NLPs. Table SII: brain regions with significant changes of RSFC between the LP and NLP groups. Figure SI: group differences of fractional anisotropy (FA), mean diffusivity (MD), radial diffusivity (RD), and axial diffusivity (AD) using voxel-based analyses in LPs compared to NLPs (AlphaSim corrected with a voxel-level threshold of *p* < 0.01 and a cluster-level threshold of *p* < 0.05). Blue and red regions denote decreased and increased DTI parameters, respectively. The numbers at the top indicate the *z* value of MNI coordinates. Figure SII: brain regions that showed altered functional connectivity with FG.R (A) and SFG.R (B), respectively, in LPs compared to NLPs. Two sample *t*-tests were performed to explore the intergroup differences. The results were corrected by the AlphaSim method at a voxel level of *p* < 0.01. All abbreviations of the brain regions are shown in Tables SI and SII.
