# Supplementary material for: Alterations of the Brain Microstructure and Corresponding Functional Connectivity in Early-Blind Adolescents
Source: Neural Plast. 2019 Feb 24;2019:2747460. doi: 10.1155/2019/2747460 (PMC6408999; doi:10.1155/2019/2747460)
Supplement: Supplementary Materials — Table SI: brain regions with significant changes in diffusion tensor imaging metrics between LPs and NLPs. Table SII: brain regions with significant changes of RSFC between the LP and NLP groups. Figure SI: group differences of fractional anisotropy (FA), mean diffusivity (MD), radial diffusivity (RD), and axial diffusivity (AD) using voxel-based analyses in LPs compared to NLPs (AlphaSim corrected with a voxel-level threshold of p < 0.01 and a cluster-level threshold of p < 0.05). Blue and red regions denote decreased and increased DTI parameters, respectively. The numbers at the top indicate the z value of MNI coordinates. Figure SII: brain regions that showed altered functional connectivity with FG.R (A) and SFG.R (B), respectively, in LPs compared to NLPs. Two sample t-tests were performed to explore the intergroup differences. The results were corrected by the AlphaSim method at a voxel level of p < 0.01. All abbreviations of the brain regions are shown in Tables SI and SII. [file 2747460.f1.pdf]

Neural Plasticity

# Alterations of the brain microstructure and corresponding functional connectivity in early blind adolescents

## Running title: Brain microstructure in EBAs.

**Zhifeng Zhou[1,2,4], Jinping Xu[2*], Leilei Shi[3], Xia Liu[1*], Fen Hou[4], Jingyi Zhou[4], Jinpei Luo[4], Qingmao Hu[2], Hengguo Li[4*]**

[1]Department of Radiology, Shenzhen Mental Health Center, Shenzhen Kangning Hospital, Shenzhen 518003, China.
[2]Institute of Biomedical and Health Engineering, Shenzhen Institutes of Advanced Technology, Chinese Academy of Sciences, Shenzhen 518055, China.
[3]Department of Mechanical Engineering, Sungkyunkwan University, Suwon 16419, South Korea.
[4]Medical Imaging Center, the First Affiliated Hospital of Jinan University, Guangzhou 510630, China.

*****Correspondence should be addressed to Xia Liu; 33342163@qq.com, Jinping Xu; jp.xu@siat.ac.cn and Hengguo Li; lhgjnu@263.net

## Supplementary Materials

**Table SI. Brain regions with significant changes in diffusion tensor imaging metrics between LPs and NLPs.**

| DTI parameters changes | Brain regions | Abbreviations | Cluster size | T score | Peak MNI | | |
|---|---|---|---|---|---|---|---|
| | | | | | x | y | z |
| FA reduction | Right Fusiform Gyrus | FG.R | 80 | -4.95 | 26 | -78 | -12 |
| | Right Middle Temporal Gyrus | MTG.R | 61 | -4.94 | 66 | -38 | -4 |
| | Right Superior Occipital Gyrus | SOG.R | 112 | -6.48 | 24 | -68 | 32 |
| FA elevation | Right Superior Frontal Gyrus | SFG.R | 107 | 4.32 | 28 | 54 | 18 |
| | Right Lingual | LING.R | 50 | 4.43 | 12 | -80 | 0 |
| MD elevation | Right Temporal Pole Middle | MTP.R | 88 | 3.93 | 26 | 12 | -38 |
| | Right Fusiform Gyrus | FG.R | 97 | 5.38 | 30 | -78 | -16 |
| MD reduction | Right Caudate | CAU.R | 67 | -3.89 | 20 | -8 | 22 |
| | Left Caudate | CAU.L | 106 | -4.15 | -18 | -8 | 26 |
| RD elevation | Right Temporal Pole Middle | MTP.R | 71 | 3.79 | 26 | 12 | -38 |
| | Right Fusiform Gyrus | FG.R | 108 | 5.24 | 30 | -78 | -16 |
| | Right Superior Occipital Gyrus | SOG.R | 83 | 7.34 | 24 | -72 | 34 |
| RD reduction | Left Caudate | CAU.L | 89 | -4.45 | -20 | -4 | 26 |
| AD elevation | Right Temporal Pole Middle | MTP.R | 93 | 4.14 | 26 | 12 | -38 |
| | Right Fusiform Gyrus | FG.R | 83 | 5.41 | 32 | -72 | -16 |
| AD reduction | Right Caudate | CAU.R | 160 | -4.31 | 18 | -10 | 22 |
| | Left Caudate | CAU.L | 87 | -4.47 | -18 | -10 | 26 |

AlphaSim corrected with a voxel-level threshold of $p < 0.01$ and a cluster-level threshold of $p < 0.05$. Abbreviations: LP, early-blind adolescents with light perception; NLP, early-blind adolescents without light perception; FA, fractional anisotropy; MD, mean diffusivity; RD, radial diffusivity; AD, axial diffusivity; and MNI, Montreal Neurological Institute.

**Table SII. Brain regions with significant changes of RSFC between LP and NLP group.**

| ROI seeds | Brain regions | Abbreviations | Cluster size (voxels) | *T* score | Peak MNI | | |
|---|---|---|---|---|---|---|---|
| | | | | | *x* | *y* | *z* |
| FG.R | Right superior frontal gyrus | SFG.R | 93 | 6.14 | 15 | 39 | -15 |
| | Right middle temporal | MTG.R | 89 | 5.41 | 63 | -51 | -6 |
| SFG.R | Left inferior temporal gyrus | ITG.L | 48 | -4.47 | -39 | -45 | -12 |
| | Right postcentral gyrus | PCG.R | 36 | -3.98 | 30 | -27 | 48 |

AlphaSim corrected with a voxel-level threshold of $p < 0.01$ and a cluster-level threshold of $p < 0.05$. Abbreviations: RSFC, resting-state functional connectivity; LP, early blind adolescents with light perception; NLP, early blind adolescents without light perception; ROI, region of interest; FG.R, right fusiform gyrus; SFG.R, right superior frontal gyrus; and MNI, Montreal Neurological Institute.

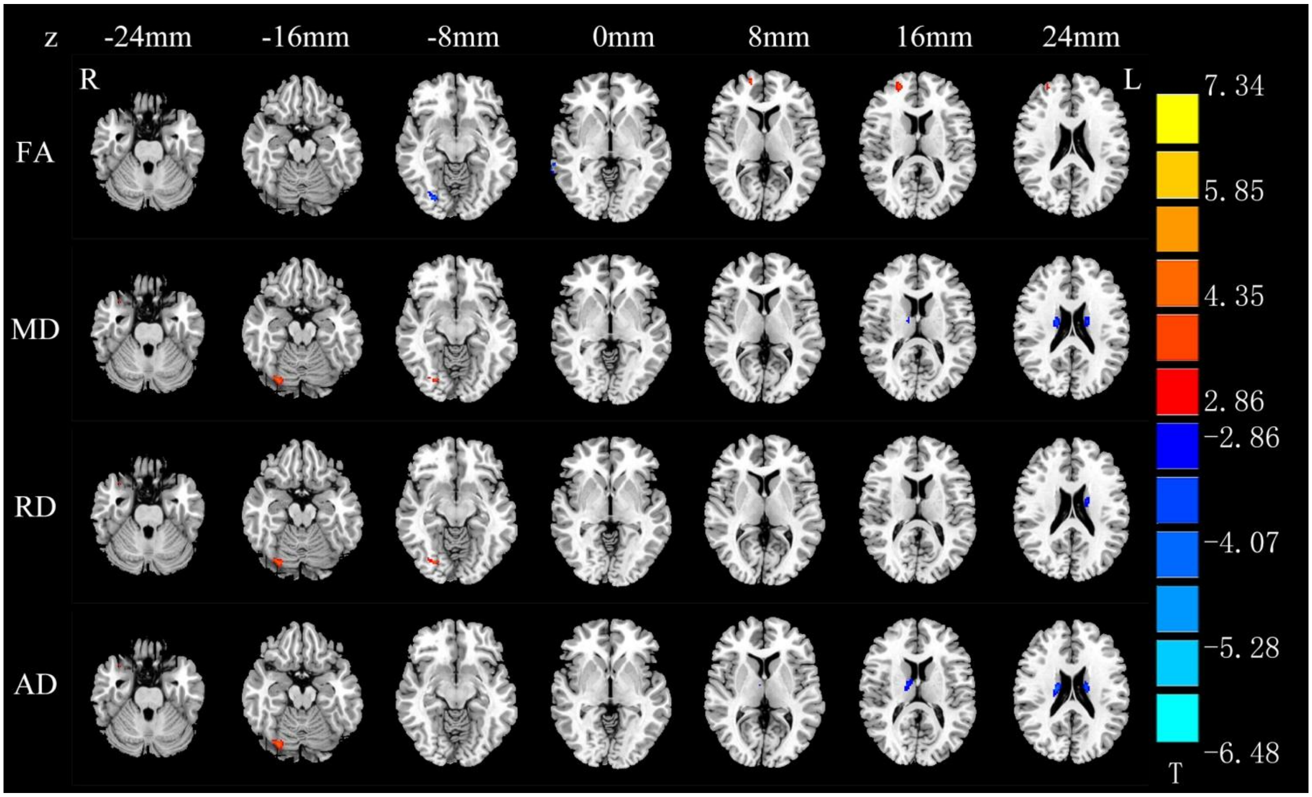


**Figure SI:** Group differences of fractional anisotropy (FA), mean diffusivity (MD), radial diffusivity (RD) and axial diffusivity (AD) using voxel-based analyses in LPs compared to NLPs (AlphaSim corrected with a voxel-level threshold of $p < 0.01$ and a cluster-level threshold of $p < 0.05$.). Blue and red regions denote decreased and increased DTI parameters, respectively. The numbers at the top indicate the z value of MNI coordinates.

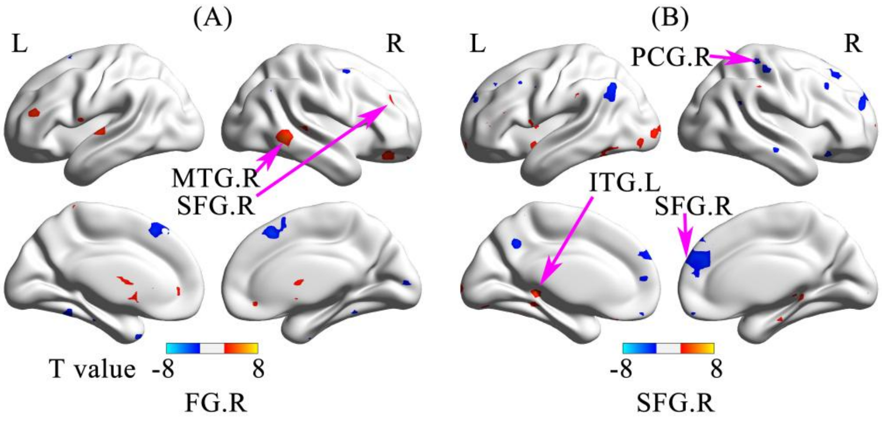


**Figure SII:** Brain regions that showed altered functional connectivity with FG.R (A) and SFG.R (B), respectively, in LPs compared to NLPs. Two sample *t*-tests were performed to explore the intergroup differences. The results were corrected by AlphaSim method at a voxel level of $p < 0.01$. All abbreviations of brain regions are shown in **Table SI-II**.

# References
